# Network-based stratification of allele-specific expression reveals patient subgroups in Huntington’s disease

**DOI:** 10.1093/bioinformatics/btag592

**Published:** 2026-08-21

**Authors:** Daan van Beek, Aishwarya Iyer, Friederike Ehrhart, Chris T Evelo, Theo M de Kok, Ilja C W Arts, Michiel E Adriaens, Martina Kutmon

**Affiliations:** Maastricht Centre for Systems Biology and Bioinformatics (MaCSBio), Maastricht University, Maastricht, the Netherlands; Maastricht Centre for Systems Biology and Bioinformatics (MaCSBio), Maastricht University, Maastricht, the Netherlands; Department of Translational Genomics, GROW/NUTRIM/MHeNs, Maastricht University, Maastricht, the Netherlands; Department of Translational Genomics, GROW/NUTRIM/MHeNs, Maastricht University, Maastricht, the Netherlands; Department of Translational Genomics, GROW/NUTRIM/MHeNs, Maastricht University, Maastricht, the Netherlands; Department of Translational Genomics, GROW/NUTRIM/MHeNs, Maastricht University, Maastricht, the Netherlands; Maastricht Centre for Systems Biology and Bioinformatics (MaCSBio), Maastricht University, Maastricht, the Netherlands; Maastricht Centre for Systems Biology and Bioinformatics (MaCSBio), Maastricht University, Maastricht, the Netherlands; Maastricht Centre for Systems Biology and Bioinformatics (MaCSBio), Maastricht University, Maastricht, the Netherlands

## Abstract

**Motivation:**

Huntington’s disease (HD) exhibits substantial variability in age of onset and disease progression that is not fully explained by CAG repeat length alone. Part of this residual variation is heritable, implicating additional genetic mechanisms. *cis*-regulatory variation, genetic variants that alter transcription and splicing of nearby genes, represents one such mechanism that can be quantified through allele-specific expression (ASE) analysis. However, methods for integrating ASE profiles into patient stratification frameworks remain underdeveloped, particularly for rare diseases with small cohorts and sparse data.

**Results:**

We adapt a network-based stratification algorithm, originally developed for somatic tumour mutations, to ASE data. By propagating gene-level ASE imbalance profiles through a protein-protein interaction network, we stratified 20 HD patients into three distinct biological patient subgroups. Differential gene expression analysis highlights neuroinflammatory pathways, including microglial activation, immune cell activation, and cytokine regulation, as key sources of inter-patient heterogeneity, while differential ASE analysis implicates proteasomal and ubiquitin-dependent protein catabolic processes, immune activation, and central nervous system development. Intersection of differentially imbalanced and expressed genes identified FAM181B as a candidate gene with potential eQTL-mediated regulation, supported by independent cis-eQTL evidence for rs3780 in the caudate and putamen, the primary HD-affected striatal regions. FAM181B encodes a nuclear protein expressed in neural tissues acting as an interactor of the Hippo pathway TEAD transcription factors, implicating transcriptional regulatory variation as a potential contributor to molecular heterogeneity between patient subgroups. Differences in cortical and striatal neuropathological scores between clusters, even when adjusted for CAG repeat length, provide clinical support for the biological relevance of the identified subgroups.

**Availability:**

All analysis code, Docker containers, and conda environments are available at https://github.com/macsbio/HD-ASE-NBS.

## 1 Introduction

Huntington’s disease (HD) is a fatal autosomal dominant neurodegenerative disorder caused by an expansion of CAG repeats in the huntingtin (HTT) gene, with a prevalence of 12 in 1 00 000 people of European descent (Nopoulos 2016). It is clinically characterized by cognitive and behavioural changes in early stages, followed by involuntary movements and progression towards more severe behavioural changes, dementia, and a complete loss of motor function (Novak and Tabrizi 2010). HTT is ubiquitously expressed and plays roles in neural tube formation, axonal transport, and synapse maintenance, but polyglutamine expansion preferentially causes neurodegeneration in the striatum and cerebral cortex through disrupted post-translational modification and protein mislocalization (Marcora and Kennedy 2010, Mccolgan and Tabrizi 2018, Lee *et al.* 2019). Despite its monogenic origin, HD exhibits striking variability in age of onset and disease progression among affected individuals. The length of uninterrupted CAG repeats accounts for approximately 60% of this variation (Lee *et al.* 2019), leaving a substantial heritable component unexplained (Djoussé *et al.* 2003, Wexler *et al*. 2004). Understanding the mechanisms underlying this residual heterogeneity is a central challenge in HD research and has direct implications for patient stratification and therapeutic development.

Transcriptional dysregulation has emerged as a key contributor to HD pathophysiology, with differential gene expression studies highlighting the role of immune response, neurodevelopmental processes, and glial cell dysfunction in both neurons and glial cells (Labadorf *et al*. 2015, 2016). However, bulk gene expression analysis captures the net outcome of regulatory activity without distinguishing between allele-specific contributions. *cis*-regulatory variation, genetic variants in regulatory regions that alter the expression of nearby genes, represents an underexplored mechanism that could account for part of the unexplained variability in HD (Gallardo-Orihuela *et al.* 2019). Unlike bulk expression changes, *cis*-regulatory effects operate at the allele level and may drive patient-specific differences in gene regulation that are invisible to standard differential expression approaches.

Allele-specific expression (ASE) analysis uses RNA-seq data to quantify allelic imbalance at heterozygous loci, where one allele is expressed at a significantly different level than the other relative to the cohort median. This serves as a direct readout of *cis*-regulatory activity, capturing the effects of expression and splicing quantitative trait loci (eQTL, sQTL) on allele-level transcription (Palacios *et al.* 2009, Pastinen 2010, Castel *et al.* 2015, Cavalli *et al.* 2016). Crucially, ASE captures regulatory signals that bulk expression analysis misses: two patients may show identical total gene expression while carrying opposing allelic imbalances driven by different *cis*-regulatory variants. We have previously validated this ASE pipeline in the context of dilated cardiomyopathy, demonstrating its capacity to uncover disease-relevant *cis*-regulatory mechanisms in a clinical cohort (van Beek *et al*. 2023), providing a foundation for its application to HD.

Patient stratification offers a powerful approach for dissecting heterogeneity in complex diseases where severity and progression vary widely among individuals. Identifying biologically meaningful patient subgroups allows the regulatory mechanisms underlying these differences to be compared directly, potentially revealing subgroup-specific drivers of disease that are masked in cohort-level analyses. In HD, where CAG repeat length alone does not fully determine clinical outcome, stratification based on *cis*-regulatory profiles offers a route to characterizing the regulatory landscape that shapes individual disease manifestation.

To achieve this, we adapt network-based stratification (NBS), originally developed for identifying tumour subtypes based on somatic mutation profiles in cancer (Hofree *et al.* 2013, Liu *et al.* 2021), to ASE data. NBS uses heat diffusion over a protein-protein interaction (PPI) network to propagate gene-level signals beyond directly affected genes, capturing broader functional consequences of molecular variation. Applying this framework to RNA-seq data from 20 HD post-mortem prefrontal cortex samples, we identify biologically distinct patient clusters and perform differential ASE and gene expression analyses between clusters to detect candidate genes and regulatory mechanisms underlying individual disease manifestation. Beyond HD, this approach is broadly applicable to other rare or complex diseases where *cis*-regulatory variation potentially contributes to phenotypic heterogeneity.

## 2 Methods

### 2.1 Dataset

A previously published HD transcriptomics dataset was obtained from the Gene expression Omnibus (GEO) data repository (GSE64810, (Labadorf *et al*. 2015, 2016)), consisting of RNA-seq data from 20 post-mortem prefrontal cortex samples, specifically from Brodmann area 9, from HD patients. Clinical data was obtained both from GEO and the corresponding publication by Labadorf *et al* (2015, 2016), including information on batch, sex, CAG repeats, age of onset, age of death, post-mortem interval, RNA integrity number, and H-V cortical and striatal scores. The H-V cortical and striatal scores are continuous measurements reflecting the degree of atrophy in those brain regions.

### 2.2 Variant calling and allele-specific expression analysis

Paired RNA-seq fastq reads for 20 HD patients were obtained using the SRA Toolkit (v3.0.0-ubuntu64) (Katz *et al.* 2022). Quality control was performed using FastQC (v0.12.0) (Wingett and Andrews 2018), followed by adapter sequence trimming using fastp (Chen *et al.* 2018). Reads were aligned to the hg19/GRCh37 reference genome using the Spliced Transcripts Alignment to a Reference (STAR) tool (v2.7.10a) in two-pass mode (Dobin *et al.* 2013). Genome indexing files were generated using the STAR genomeGenerate function with the hg19/GRCh37 assembly FASTA file, corresponding Ensembl genome annotation. Heterozygous positions were identified using the 1000 Genomes Project Phase 3 (build 37) VCF file containing 38.3 million biallelic loci (Auton *et al.* 2015, Dyer *et al.* 2025). The resulting BAM files were prepared for downstream analysis using GATK tools, including AddOrReplaceReadGroups and SplitNCigarReads functions, respectively (Mckenna *et al.* 2010).

To generate VCF files per sample, the HaplotypeCaller function from GATK tool was implemented. Variants were filtered to retain only biallelic single nucleotide polymorphisms (SNPs). The filtered VCF files for each sample were then used as input for correcting reference allele mapping bias using WASP (v0.3.4) (van de Geijn *et al*. 2015, Asiimwe and Dobin 2024). WASP identifies and removes reads whose alignment is biased towards the reference allele at heterozygous sites. This step is critical for accurate ASE quantification, as standard aligners systematically favour reads matching the reference genome. Only WASP-corrected BAM files were used for counting allelic imbalance. ASE analysis was conducted using GATK ASEReadCounter on the WASP-corrected BAM files. For each sample, ASEReadCounter tallied the number of reads overlapping heterozygous SNP positions, separately counting reference and alternative allele-supporting reads (Mckenna *et al.* 2010).

Patient-specific allelic imbalance profiles were determined from the ASEReadCounter output following the pipeline described in van Beek *et al* (2023), which quantifies the relative abundance of alleles at heterozygous loci as a proxy for *cis*-regulatory variation. Allelic imbalance was quantified on a scale from 0.5 to 1, where 0.5 indicates balanced expression of both alleles, and 1 indicates monoallelic expression of either the reference or alternative allele. Significant allelic imbalance was defined as *q*-value < 0.05, based on a binomial test where the expected probability of observing each allele equals the cohort median ASE, and the observed probability is the individual’s measured ASE score at each locus. Only heterozygous sites with a minimum combined read depth of 10 reads (≥10 counts total across both alleles) were included in the analysis. Additionally, loci with imbalance scores below the population median were excluded to further account for residual mapping biases (Castel *et al.* 2015).

Finally, raw gene-level read counts were generated from the BAM files using featureCounts (v2.1.1) (Liao *et al.* 2014) for subsequent differential expression and network-based stratification analyses. The protein-coding genes using the biomaRt package (v2.58.2) (Durinck *et al.* 2009) in R (v4.3.3) (R Core Team 2025) with a mean count of ≥ 10 across samples and expressed in at least 20% of samples were used for further downstream analyses.

### 2.3 Network-based stratification on allele-specific expression data

A network-based stratification algorithm was applied to cluster patients based on their allelic imbalance profile. Originally developed for tumour mutation data (Hofree *et al.* 2013, Huang *et al*. 2018), the pyNBS method requires a binary matrix assigning the presence or absence of a certain mutation in a gene per patient. The scores of these genes are then distributed to neighboring genes in a PPI network through heat diffusion, to simulate the downstream effect of the mutation in the context of protein interactions. The ASE data was adapted to work with network-based stratification by creating subject-specific binary profiles, where the presence of at least one significantly imbalanced locus in a gene marks it as imbalanced, assigning it a score of 1, and otherwise assigning it a score of 0. These profiles were mapped onto the human STRING (v12.0) (Szklarczyk *et al.* 2023) PPI network created with a confidence score of > 0.9. The pyNBS algorithm then performed heat diffusion to simulate the spread of the effect of each gene imbalance, and subsequently clustered patients based on the similarity of their smoothed network profiles over 1000 iterations. The similarity between patients was determined using the co-cluster frequency, indicating how often a pair of patients clustered together across iterations. Thus, a cluster represents a group of patients with similarly affected PPI neighbourhoods through *cis*-regulatory events.

To assess the added value of network propagation, k-means clustering was applied to batch-corrected gene expression data and the raw binary ASE imbalance matrix as baseline comparisons. Neither approach identified stable patient subgroups, as shown by the absence of block structure in pairwise Pearson correlation matrices and no clear optimal cluster number in gap statistic analyses across *k* = 1–10 (Fig. S1, available as supplementary data at *Bioinformatics* online), supporting the necessity of network-based propagation for meaningful ASE-based patient stratification.

### 2.4 Comparative analysis of patient clusters

Differences in allelic imbalance and clinical parameters across the three clusters were assessed using the Kruskal-Wallis test (Kruskal and Wallis 1952, R Core Team 2025), with effect sizes estimated using epsilon-squared ($\epsilon^{2}$). For the allelic imbalance analysis, loci with fewer than three observations in any of the three clusters were excluded. Given the small sample size inherent to rare disease cohorts (total *n* = 20), a nominal *P*-value < 0.05 was used as the significance threshold to select significantly imbalanced loci, as multiple testing correction using Benjamini-Hochberg false discovery rate resulted in no significant findings. A gene was considered differentially imbalanced if at least one of its loci reached significance. Significantly differentially imbalanced loci were subsequently annotated using a reference annotation dataset to facilitate downstream biological interpretation. For the clinical variables analysis, variables tested included CAG repeats, age of onset, age of death, post-mortem interval, and RNA integrity number, with variables containing fewer than three observations per cluster excluded from analysis. Multiple testing correction was applied using the Benjamini-Hochberg false discovery rate, and results were visualized with nominal *P*-values and ($\epsilon^{2}$) effect sizes annotated on the plots.

For the differential gene expression analysis, raw counts were restricted to protein-coding genes passing the expression filters described in Section Variant calling and allele-specific expression analysis. Batch correction was performed using the sva R-package (v3.50.0) (Leek *et al.* 2012). The batch-corrected raw counts were analysed using the DESeq2 R-package (v1.42.1) (Love *et al.* 2014) with the likelihood ratio test (LRT), comparing a full model with cluster as the sole factor against an intercept-only reduced model, enabling the identification of genes whose expression significantly differs across the three clusters. The counts were then transformed for downstream visualization. Given the small sample size inherent to rare disease cohorts (total *n* = 20), a nominal *P*-value < 0.05 was used as the significance threshold, as multiple testing correction resulted in no significant findings.

The clusterProfiler R-package (v4.10.1) (Yu *et al.* 2012) was used to perform Gene Ontology (GO) enrichment analysis for biological processes with lists of the differentially expressed or imbalanced genes. Default parameters of the enrichGO() function were applied with the human genome (org.Hs.eg.db) (Carlson 2025) as the background universe.

### 2.5 Open-source code

All computational analyses in this study are fully reproducible. Tool-specific Docker containers, analysis scripts, and conda environment files are publicly available via GitHub, enabling reproduction of results and adaptation for further exploration (https://github.com/macsbio/HD-ASE-NBS).

## 3 Results

### 3.1 ASE analysis shows widespread and diverse allelic imbalance

Raw count data obtained from a public transcriptomics dataset of 20 HD patients was pre-processed using our ASE pipeline (see Section 2 for details). The analysis initially yielded allelic-specific count data for 797 249 genomic loci across 20 patients. After filtering for loci with read counts > 10 for both alleles, 156 535 loci remained. Subsequently, 69 950 loci were identified with an ASE score higher than the population median (0.58), which was used to correct for mapping bias. Each patient had a median of 11 739 loci measured, with a range of 8079 to 15 751 loci. When focusing on the 3486 loci detected in at least 50% (*n* = 10) of the patients, loci in two genes (FCF1 and ACTN2) showed allele-specific imbalance in at least 75% (*n* = 15) of participants.

### 3.2 ASE-based stratification informed by protein-protein interactions reveals three clusters

**Figure 1:**
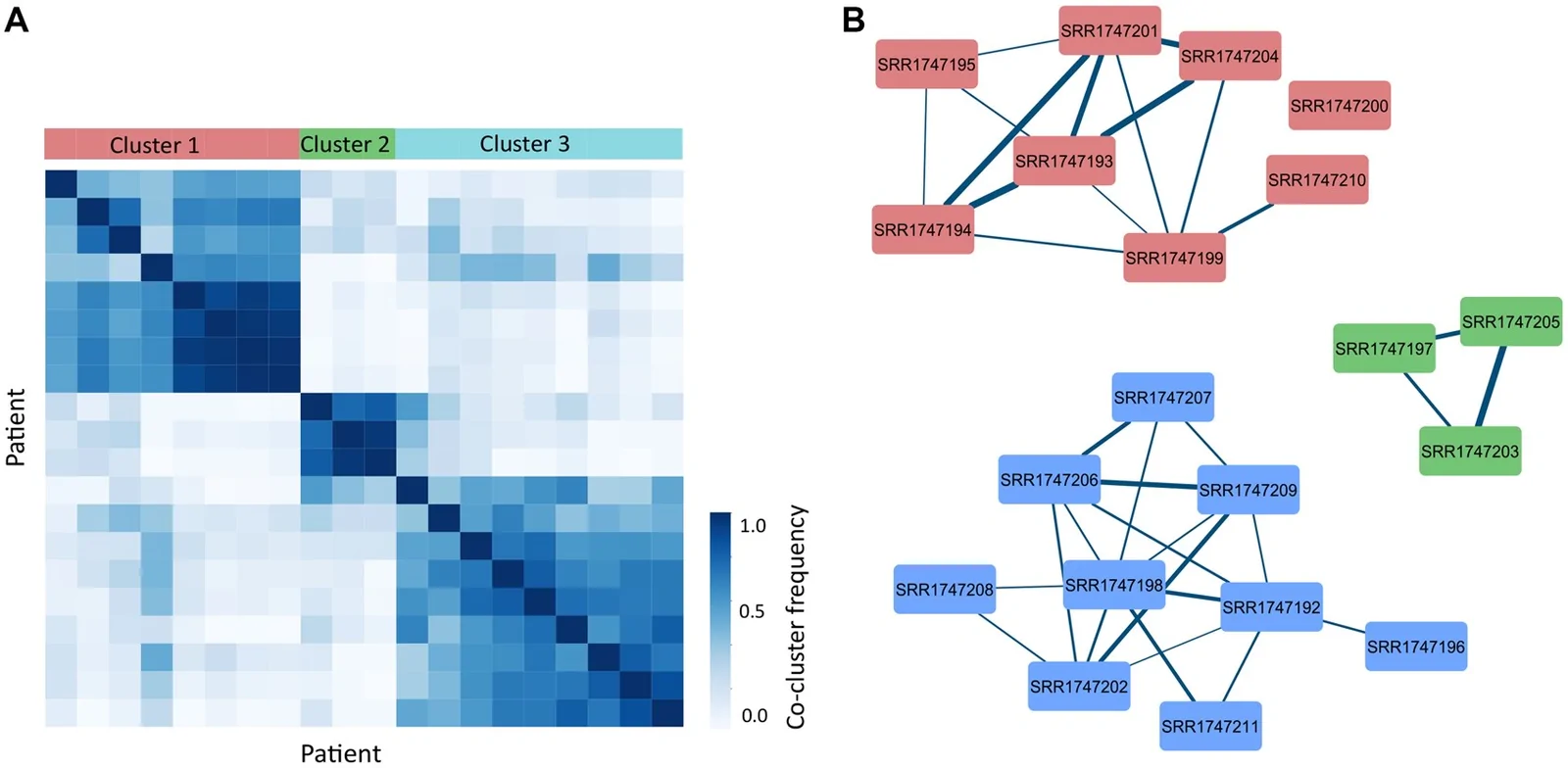
Patient clusters identified by network-based stratification of allele-specific expression profiles. (A) The heatmap shows the co-cluster frequency for each patient pair over 1000 iterations. Patients are grouped by cluster assignment, indicated by coloured bars (red: cluster 1, green: cluster 2, cyan: cluster 3). (B) Network representation of patient clusters. Nodes represent individual patients, coloured by cluster assignments. Edges connect patients with a co-cluster frequency > 0.63, with edge width scaling linearly with co-cluster frequency.

We implemented a gene-based analysis using ASE data, taking into account functional relationships between genes as informed by PPIs. An imbalanced gene was defined as having at least one significantly imbalanced locus in that gene for a particular patient. These genes receive a score of 1 in the PPI network, and all other genes receive a score of 0. We used the main connected component of the human STRING PPI network, consisting of 9418 nodes and 74 288 edges. In total, there were 2760 unique genes with a detected ASE event in any of the patients, of which 1314 were also present in the main network component. After heat diffusion of the gene scores, the network-based stratification algorithm identified three patient clusters: cluster 1 (red, *n* = 8), cluster 2 (green, *n* = 3), cluster 3 (cyan, *n* = 9), see Fig. 1. The quality and stability of the three-cluster solution were assessed using silhouette analysis and consensus clustering, confirming good overall cluster separation (mean silhouette width (≈0.57)) and high within-cluster consensus relative to between-cluster consensus across all three clusters (Fig. S2, available as supplementary data at *Bioinformatics* online). To assess robustness of the three-cluster solution, a leave-one-out stability analysis was performed by removing each patient in turn and re-clustering the remaining 19 patients with *k* = 3. A mean Adjusted Rand Index (ARI) of 0.981 was observed, with 17 of 20 patients yielding ARI = 1.00 upon removal, indicating very good cluster stability (Fig. S3, available as supplementary data at *Bioinformatics* online). All three cluster 2 patients consistently co-clustered upon removal of any one, confirming cluster 2 is not driven by a single outlier. Minor instability was observed for two cluster 3 patients (SRR1747198 and SRR1747202, ARI = 0.869), consistent with their borderline silhouette values (Fig. S2, available as supplementary data at *Bioinformatics* online).

When focusing on loci with an ASE score above the population median, the median number of detected loci was comparable: 5115 in cluster 1, 4247 in cluster 2, and 4228 in cluster 3. However, the number of significantly imbalanced loci (*q*-value < 0.05) was notably higher in cluster 1, with a median of 257 loci per patient, compared to 40 in cluster 2 and 84 in cluster 3. This pattern is consistent with the ASE distributions, where cluster 1 shows a heavier upper tail, indicating more frequent or stronger imbalance events (Fig. S4, available as supplementary data at *Bioinformatics* online).

### 3.3 Cluster comparisons of differential imbalance and expression

To further investigate regulatory differences between the three patient clusters, we performed differential allelic imbalance and differential gene expression analyses.

#### 3.3.1 Differential allelic imbalance analysis

To assess the differential imbalance between clusters, we applied the Kruskal-Wallis test to loci with at least three ASE measurements across all clusters. In total, 36 genes showed differential imbalance in at least one locus (*P*-value < 0.05). GO enrichment analysis on these genes revealed five biological themes (Fig. S5A, available as supplementary data at *Bioinformatics* online). First, proteasomal and ubiquitin-dependent protein catabolic processes were prominently enriched, including proteasome-mediated ubiquitin-dependent protein degradation, regulation of proteasomal protein catabolic process, and the ERAD pathway. Second, immune activation processes were enriched, including T cell activation, lymphocyte activation, and leukocyte activation. Third, apoptotic and cell death signalling processes were represented, including intrinsic apoptotic signalling, signal transduction by p53 class mediator, and regulation of programmed cell death. Fourth, developmental processes were enriched, including brain development and central nervous system development. Finally, response to endoplasmic reticulum stress and regulation of cellular response to stress were enriched, suggesting broader proteostatic dysregulation across clusters.

### 3.3.2 Differential gene expression analysis

Differential gene expression analysis identified 723 differentially expressed genes across the three clusters (*P*-value < 0.05). GO enrichment analysis highlighted five biological themes (Fig. S5B, available as supplementary data at *Bioinformatics* online). First, immune activation and adaptive immunity processes were prominent, including T-cell, lymphocyte, and leukocyte activation and proliferation, leukocyte cell-cell adhesion, and antigen processing and presentation via MHC class II. Second, innate immune processes were enriched, including granulocyte and neutrophil activation, leukocyte migration, and regulation of acute inflammatory response. Third, neuroinflammatory processes were represented, including microglial activation and astrocyte development. Fourth, cytokine signalling was enriched, represented by IL6 production and related processes. Finally, protein synthesis-related processes, including ribosome biogenesis and cytoplasmic translation, were enriched, suggesting broad transcriptional dysregulation beyond immune pathways.

### 3.3.3 FAM181B suggests eQTL-driven dysregulation

**Figure 2:**
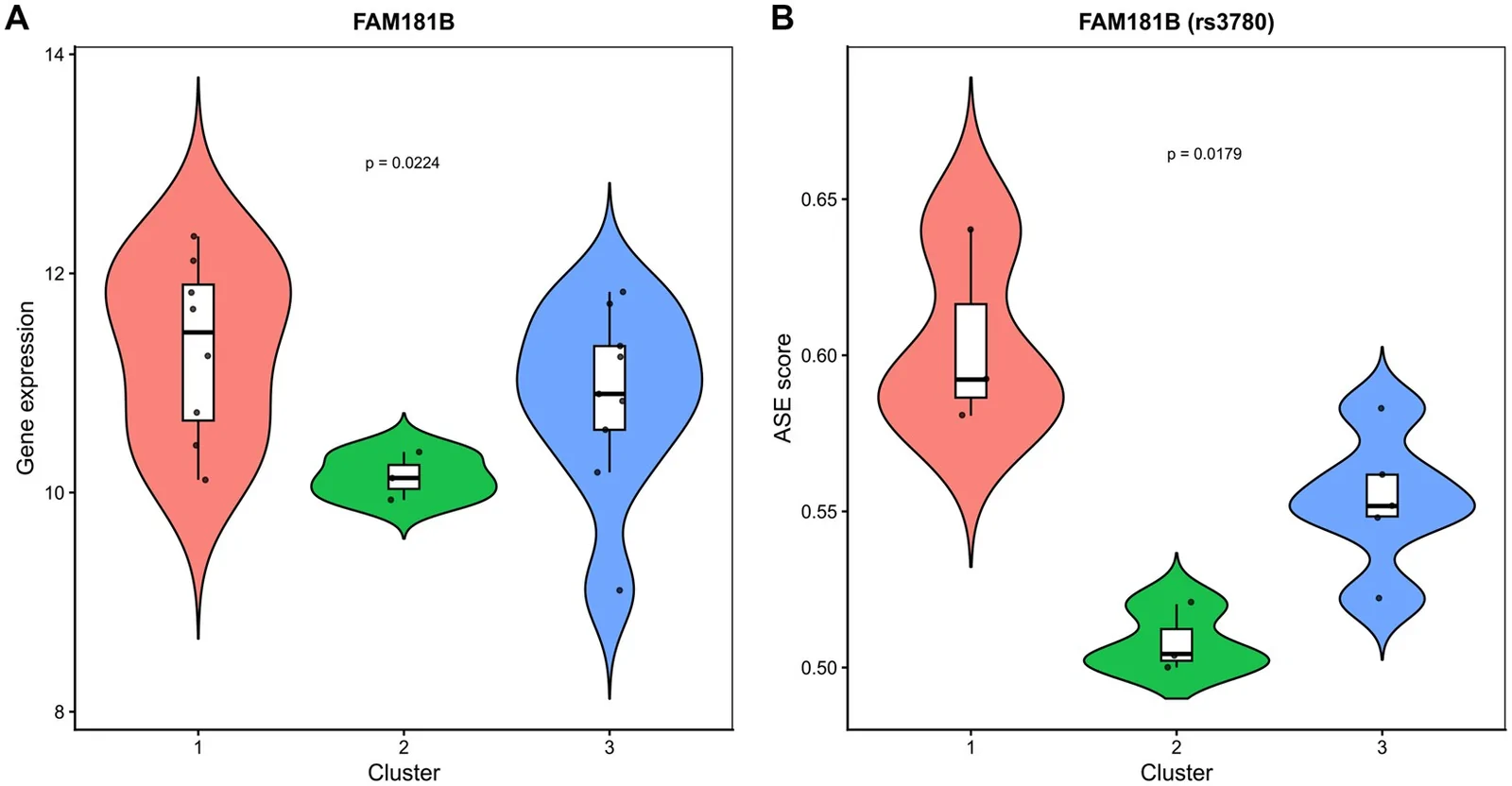
Differential allelic imbalance and gene expression of FAM181B across the three patient clusters. (A) Violin plot showing log-transformed gene expression values (*y*-axis) across clusters (*x*-axis) for FAM181B (*P* = 0.0224). (B) Violin plot showing ASE scores at locus rs3780 within FAM181B across clusters (*P* = 0.0179). Each violin includes a boxplot showing the median and interquartile range, with individual data points shown as jittered dots. Nominal *P*-values from the Kruskal-Wallis test are annotated. Clusters are colour-coded: cluster 1 (red, *n* = 8), cluster 2 (green, *n* = 3), cluster 3 (blue, *n* = 9).

We identified one gene at the overlap of differential imbalance and differential expression between the three clusters (*P*-value < 0.05): FAM181B (see Fig. 2). The concurrent differential imbalance at locus rs3780 (*P* = 0.0179, $\epsilon^{2}$ = 0.805) and differential expression (*P* = 0.0224) of FAM181B, without allelic dosage compensation, suggests an underlying eQTL regulatory mechanism in which a genetic variant drives differences in overall gene expression levels.

### 3.3.4 Clinical variables association analysis

**Figure 3:**
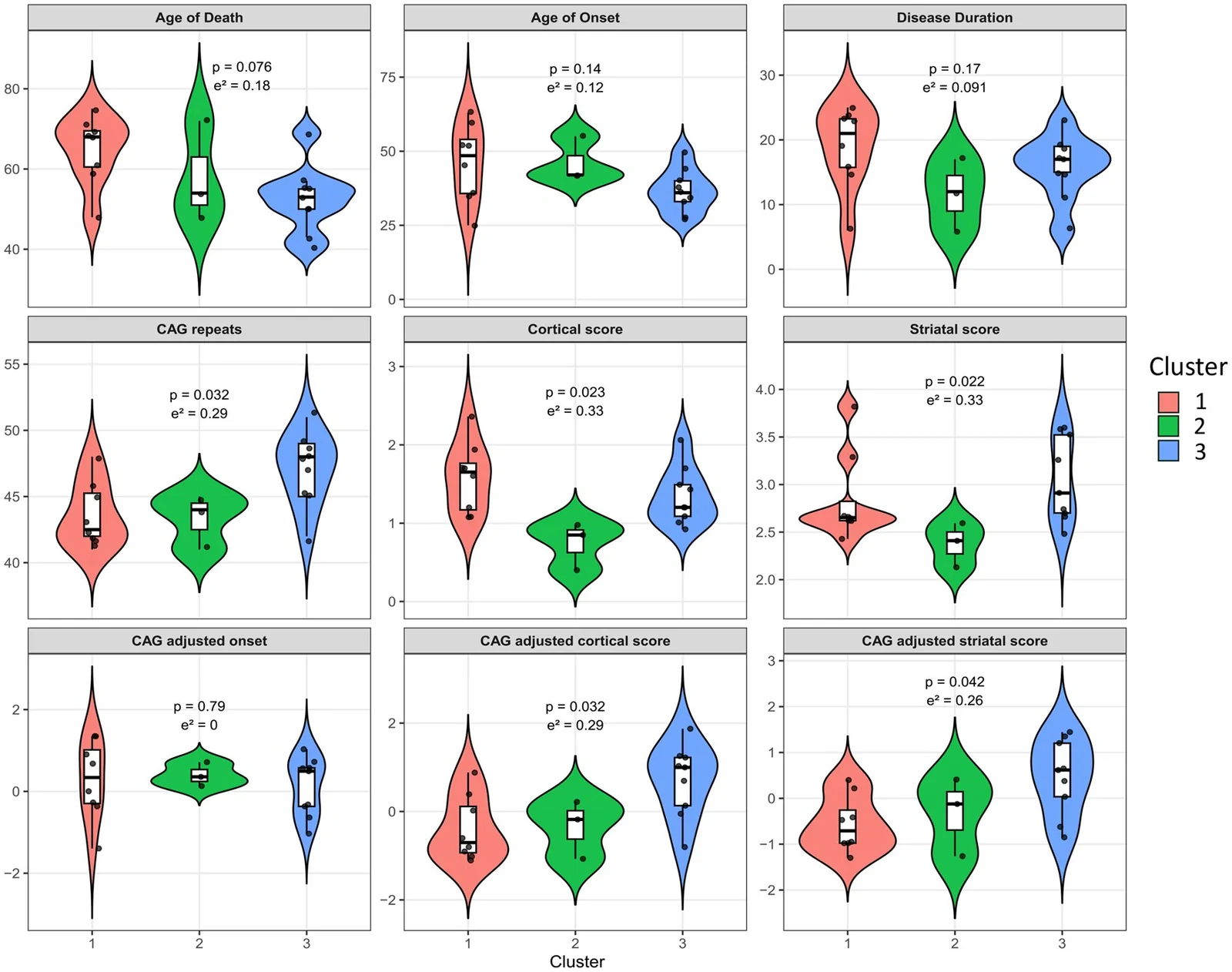
Association of clinical variables with patient clusters. Violin plots showing the distribution of clinical parameters across the three patient clusters identified by network-based stratification. Variables shown include age of death, age of onset, disease duration, CAG repeats, cortical score, striatal score, CAG-adjusted onset, CAG-adjusted cortical score, and CAG-adjusted striatal score. Each violin plot shows the median and interquartile range. Nominal *P*-values and epsilon-squared ($\epsilon^{2}$) effect sizes from the Kruskal-Wallis test are annotated for each variable. Clusters are colour-coded: cluster 1 (red, *n* = 8), cluster 2 (green, *n* = 3), cluster 3 (blue, *n* = 9).

The association between the three clusters and clinical parameters was assessed using the Kruskal-Wallis test (Fig. 3). CAG repeat length differed significantly across clusters (*P* = 0.032, $\epsilon^{2}$ = 0.29), with cluster 1 displaying lower CAG repeat counts compared to clusters 2 and 3. Unadjusted cortical score (*P* = 0.023, $\epsilon^{2}$ = 0.33) and striatal score (*P* = 0.022, $\epsilon^{2}$ = 0.33) were both significantly different across clusters, with cluster 2 showing lower scores. However, after adjusting for CAG repeat length, this pattern shifted: CAG-adjusted cortical score (*P* = 0.032, $\epsilon^{2}$ = 0.29) and CAG-adjusted striatal score (*P* = 0.042, $\epsilon^{2}$ = 0.26) were lowest in cluster 1 and highest in cluster 3, suggesting CAG-independent factors contribute to neuropathological severity across clusters.

Age of death (*P* = 0.076, $\epsilon^{2}$ = 0.18), age of onset (*P* = 0.14, $\epsilon^{2}$ = 0.12), and disease duration (*P* = 0.17, $\epsilon^{2}$ = 0.091) did not reach statistical significance, though moderate effect sizes were observed, with cluster 1 showing trends towards later age of onset, longer disease duration, and later age of death. CAG-adjusted onset did not differ across clusters (*P* = 0.79, $\epsilon^{2}$ = 0).

Post-mortem interval (*P* = 0.056, $\epsilon^{2}$ = 0.24) and RNA integrity number (*P* = 0.75, $\epsilon^{2}$ = 0) did not differ significantly across clusters, suggesting that the observed expression differences are unlikely to be driven by sample quality or post-mortem degradation (Fig. S6, available as supplementary data at *Bioinformatics* online).

## 4 Discussion

In this study, we applied network-based stratification to ASE data to identify subgroups in HD, revealing three clusters with distinct *cis*-regulatory profiles and differences in neuropathological severity. By adapting a framework originally developed for somatic mutation profiles in cancer to *cis*-regulatory variation in a neurodegenerative disease, we demonstrate that ASE-derived gene imbalance profiles, integrated with PPI networks, can be utilized to stratify patients into biologically distinct subgroups. The identification of clusters that differ in CAG-adjusted neuropathological scores, neuroinflammatory gene expression, and candidate eQTL gene suggests that *cis*-regulatory heterogeneity contributes independently to variation in HD disease manifestation, complementing both the well-established role of CAG repeat length and previous evidence implicating epigenetic and transcriptional regulation in HD variability.

### 4.1 Patient stratification and clinical correlates

The network-based stratification identified three clusters with distinct allelic imbalance profiles and differing clinical characteristics. Cluster 1, comprising eight patients, was characterized by lower CAG repeat counts compared to clusters 2 and 3. Trends towards later age of onset, longer disease duration, and later age of death, while not reaching statistical significance, are consistent with a milder disease course in this subgroup. Notably, after adjusting for CAG repeat length, cluster 1 showed the lowest neuropathological scores, suggesting that CAG-independent factors contribute to its relatively preserved neuropathological profile. By contrast, cluster 3 showed the highest CAG-adjusted cortical and striatal scores, indicating greater neuropathological damage than predicted by CAG repeat length alone. Cluster 2, comprising only three patients, showed the lowest unadjusted neuropathological scores. However, the small size of cluster 2 substantially limits the conclusions that can be drawn, and validation in larger independent cohorts is needed to confirm the stability and biological relevance of all three subgroups.

### 4.2 Neuroinflammatory signatures across clusters

A consistent neuroinflammatory signal emerged independently from both the differential ASE and differential gene expression analyses, strengthening its biological relevance. GO enrichment of differentially imbalanced genes revealed processes related to immune activation, including T cell activation, lymphocyte activation, and leukocyte activation, as well as proteasomal and ubiquitin-dependent protein catabolic processes, apoptotic signalling, and brain and central nervous system development.

Independently, differential gene expression analysis identified enrichment of immune cell activation, leukocyte migration, microglial activation, astrocyte development, and cytokine signalling pathways. The convergence of both analyses on neuroinflammatory processes as a key differentiator between clusters is consistent with the established role of neuroinflammation and glial dysregulation in HD pathophysiology (Silvestroni *et al.* 2009, Crotti and Glass 2015, Labadorf *et al*. 2015, 2016), and suggests that *cis*-regulatory variation may contribute to inter-patient differences in neuroinflammatory state.

### 4.3 Candidate eQTL gene and HD relevant pathways

By intersecting differential imbalance and differential expression analyses, we identified FAM181B as a candidate gene where *cis*-regulatory variation appears to alter overall gene expression, consistent with eQTL-mediated regulation.

FAM181B has been identified as a novel interactor of TEAD transcription factors, the nuclear effectors of the Hippo signalling pathway, binding via an Ω-loop similarly to YAP and TAZ, with full-length FAM181B confirmed to interact with TEAD in cells (Bokhovchuk *et al.* 2020). In murine development, Fam181b shows strong expression in the ventricular zone of the forebrain, midbrain, and neural tube, with the highest expression levels maintained in the cerebrum and cerebellum in adult tissues, and transcripts enriched in the transcriptome of astrocytes (Marks *et al.* 2016).

Cross-referencing the differentially imbalanced rs3780 locus with GTEx Analysis Release V10 eQTL data (Lonsdale *et al.* 2013) revealed that rs3780 has been independently reported as a significant cis-eQTL for FAM181B in two HD-relevant striatal brain regions: the caudate (basal ganglia, $P=2.6\times 10^{-6}$, NES = −0.16) and the putamen (basal ganglia, $P=6.8\times 10^{-5}$, NES = −0.13). The caudate and putamen together constitute the striatum, the primary site of neurodegeneration in HD, providing additional evidence that the differential expression may be eQTL-driven rather than a secondary consequence of neurodegeneration.

Additional independent support for allele-specific regulatory variation at FAM181B comes from a recent epigenome editing study demonstrating that the FAM181B promoter contains a methylated CpG island harbouring a SNP that enables allele-specific discrimination, with stable allele-specific demethylation achieved at this locus using a TET1-dCas9 editing approach (Rajaram *et al.* 2024). This provides a mechanistic basis for the allele-specific expression signal observed at rs3780, consistent with differential promoter methylation between alleles as a plausible driver of the cis-regulatory imbalance identified across patient clusters.

### 4.4 Methodological considerations and limitations

Despite the low sample size inherent to HD and rare diseases in general, the results provide further insight into disease mechanisms and variation among affected patients. The network-based stratification framework is well-suited to the sparse data characteristic of rare disease cohorts. It is important to note that the small size of cluster 2 (*n* = 3) in particular limits the strength of conclusions that can be drawn for this subgroup, and validation in larger independent cohorts remains an important next step.

While locus-level clustering would retain splicing-specific imbalances, gene-level analysis captures functional *cis*-regulatory variation at a resolution appropriate for network integration. By incorporating PPI interactions, the framework co-analyses genes within the same network neighbourhood, capturing downstream effects of *cis*-regulatory variation that isolated gene-level analysis would miss, and providing biological interpretability beyond simple clustering.

The key analytical choices were made to balance sensitivity and robustness. Defining a gene as imbalanced based on at least one significantly imbalanced locus deliberately retains sQTL-derived variation. Restricting differential imbalance analysis to loci with at least three measurements per cluster and at least 10 reads per allele ensures reliable imbalance detection with reduced technical noise. Using the population median ASE score as baseline for binomial models provides a correction for mapping bias, with the tradeoff of raising the imbalance threshold for reads with no mapping bias. The population median ASE score (0.58) serves as a population-wide null for the binomial test, shifting the significance boundary uniformly across all patients when varied. Since stratification is driven by differential ASE patterns between patients rather than absolute ASE levels, a global threshold shift largely cancels at the level of between-patient contrasts, making cluster assignments robust to moderate variation around this value. A per-individual median would be an alternative, but is itself unstably estimated in a cohort of *n* = 20, making the population median the more reproducible choice. To our knowledge, allelic imbalance-based clustering to find patterns of *cis*-regulatory variation has not yet been applied to HD data, and the pipeline developed here serves as an example of how existing rare disease data can be leveraged to explore disease heterogeneity in new ways without necessitating new data collection.

In the future, combining whole-genome sequencing with deep, long-read RNA sequencing may enable drawing more direct causal connections between genomic variations and allelic imbalance.

## 5 Conclusion

This study demonstrates that network-based stratification of ASE profiles can reveal biologically meaningful patient subgroups in HD, capturing *cis*-regulatory heterogeneity that is not explained by CAG repeat length alone. The identification of proteasomal dysregulation, immune activation, and apoptotic signalling through differential ASE analysis, converging with neuroinflammatory signatures from differential gene expression, points to cis-regulatory variation as a contributor to inter-patient differences in HD pathobiology independent of CAG repeat length. The candidate eQTL gene FAM181B, supported by independent striatal eQTL evidence and allele-specific promoter methylation, represents a hypothesis-generating finding pointing to potential CAG-independent disease mechanisms worthy of further investigation. Beyond HD, the framework presented here offers a broadly applicable approach for regulatory subtype discovery in rare and complex diseases.

## Supplementary Material

btag592_Supplementary_Data

## Data Availability

The data underlying this article are available in the Gene Expression Omnibus (GEO) repository, under accession number GSE64810 (https://www.ncbi.nlm.nih.gov/geo/query/acc.cgi?acc=GSE64810). All analysis scripts, Docker containers, and conda environment files supporting the reproducibility of this work are available on GitHub (https://github.com/macsbio/HD-ASE-NBS).
