# Supplementary material for: Network-based stratification of allele-specific expression reveals patient subgroups in Huntington’s disease
Source: Bioinformatics. 2026 Aug 21;42(Suppl 2):btag592. doi: 10.1093/bioinformatics/btag592 (PMC13501290; doi:10.1093/bioinformatics/btag592)
Supplement: Supplementary file 1 [file btag592_supplementary_data.pdf]

Supplementary figures for

Article Title: Network-based stratification of allele-specific expression reveals patient subgroups in Huntington's disease

Authors: Daan van Beek, Aishwarya Iyer, Friederike Ehrhart, Chris T.Evelo, Theo M.de Kok, Ilja C.W.Arts, Michiel Adriaens and Martina Kutmon.

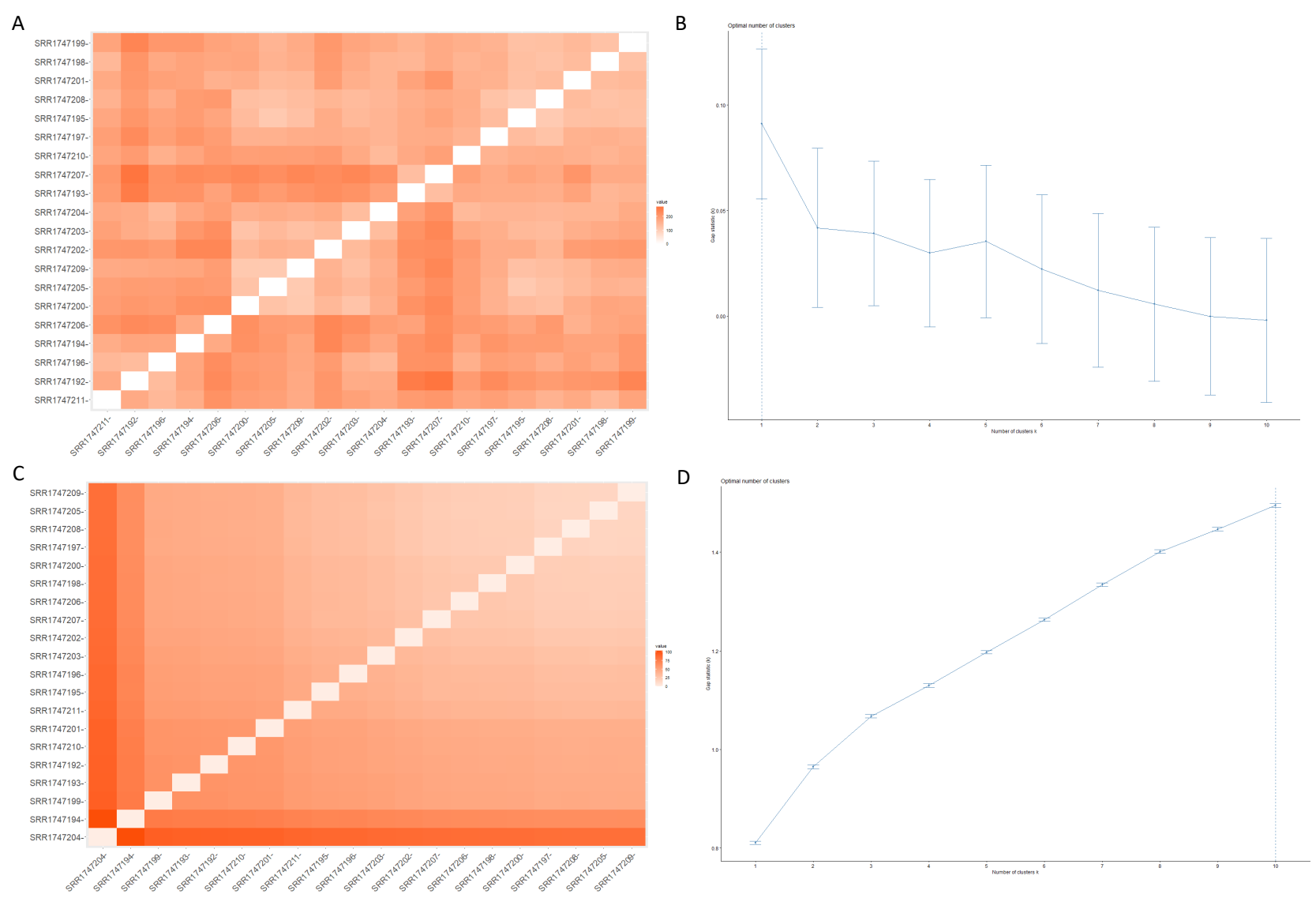

Supplementary Figure 1: Cluster quality assessment for baseline patient stratification models. (A) Pairwise sample correlation matrix of batch-corrected gene expression data across all patients. Colour scale indicates Pearson correlation between patient pairs. No block structure is apparent, indicating no stable patient subgroups emerge from gene expression data alone. (B) Gap statistic analysis across a range of cluster numbers ($k = 1$–10) for gene expression data, showing no clear optimal k, confirming the absence of meaningful cluster structure. (C) Pairwise sample similarity matrix calculated directly from the raw binary ASE imbalance profiles across all patients. Colour scale indicates pairwise similarity between patient pairs. No block structure is observed, indicating that raw allelic imbalance profiles alone are insufficient for patient stratification. (D) Gap statistic analysis across a range of cluster numbers ($k = 1$–10) for the raw binary ASE data, identifying $k = 1$ as the optimal number of clusters, confirming the absence of meaningful structure.

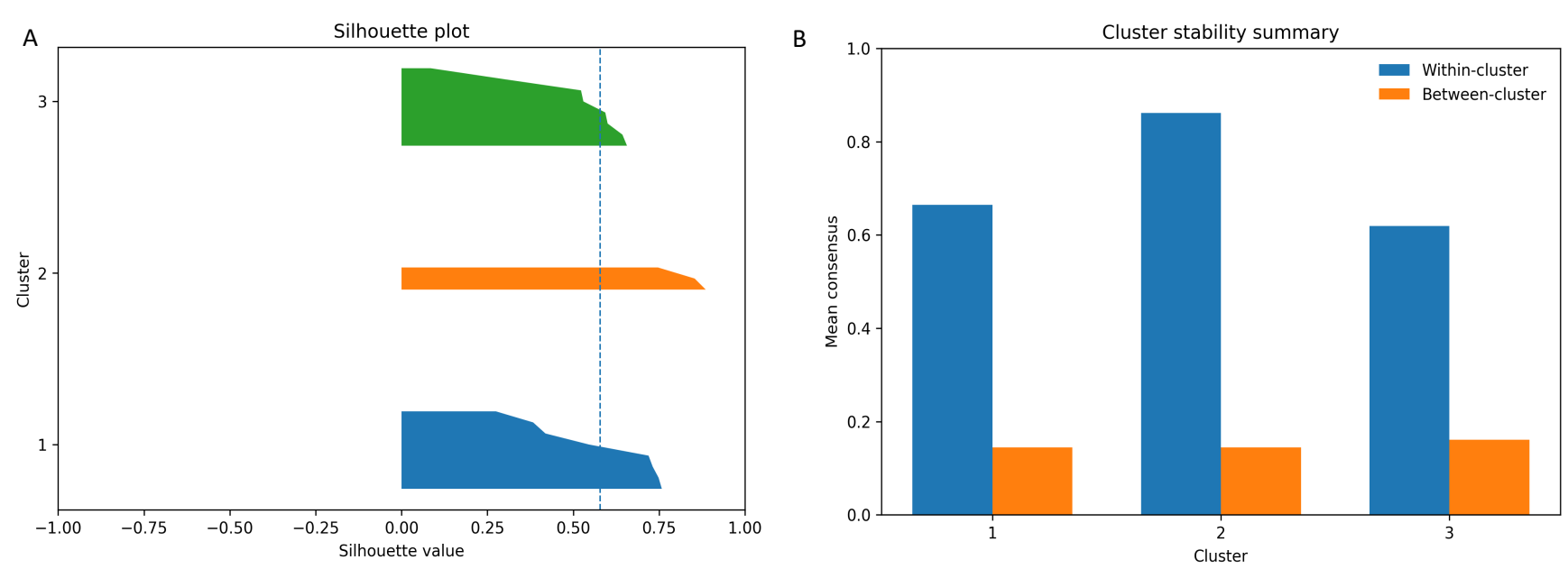

Supplementary Figure 2: Cluster quality and stability assessment of the network-based stratification solution. A) Silhouette plot showing the silhouette values for each patient across the three clusters. Each horizontal bar represents one patient, colored by cluster assignment (blue: cluster 1, orange: cluster 2, green: cluster 3). The dashed vertical line indicates the mean silhouette width across all patients (≈ 0.57), reflecting overall good cluster separation. Positive silhouette values indicate that patients are well-matched to their assigned cluster and poorly matched to neighboring clusters. B) Cluster stability summary bar plot showing mean within-cluster consensus (blue) and mean between-cluster consensus (orange) for each of the three clusters. Higher within-cluster consensus relative to between-cluster consensus indicates stable, well-separated clusters. Cluster 2 shows the highest within-cluster consensus (≈ 0.85).

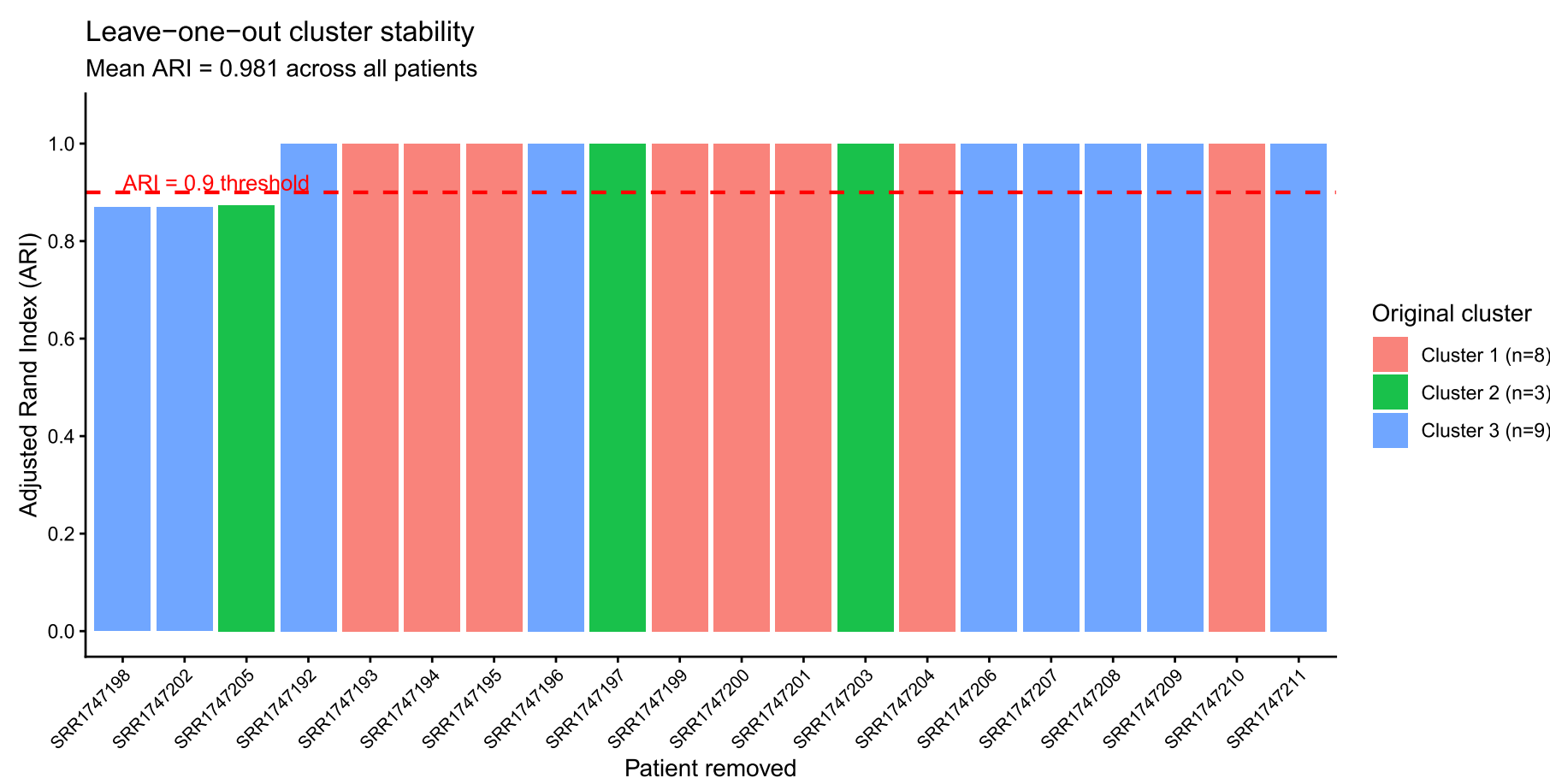

Supplementary Figure 3: Leave-one-out cluster stability analysis. Bar plot showing the Adjusted Rand Index (ARI) for each of the 20 leave-one-out iterations. In each iteration, one patient was removed from the co-cluster frequency matrix and the remaining 19 patients were re-clustered using hierarchical clustering with $k = 3$. The ARI was calculated by comparing the new cluster assignments to the original network-based stratification solution. Bars are coloured by the original cluster assignment of the removed patient: cluster 1 (red, $n = 8$), cluster 2 (green, $n = 3$), and cluster 3 (blue, $n = 9$). The red dashed line indicates an ARI threshold of 0.9. A mean ARI of 0.981 was observed across all patients, with 17 of 20 patients yielding ARI = 1.00 upon removal, indicating near-perfect cluster preservation. Removal of any of the three cluster 2 patients consistently resulted in the remaining two patients co-clustering together, demonstrating that cluster 2 is not driven by a single outlier patient. Minor instability was observed for two cluster 3 patients (SRR1747198 and SRR1747202, ARI = 0.869), consistent with their borderline silhouette values.

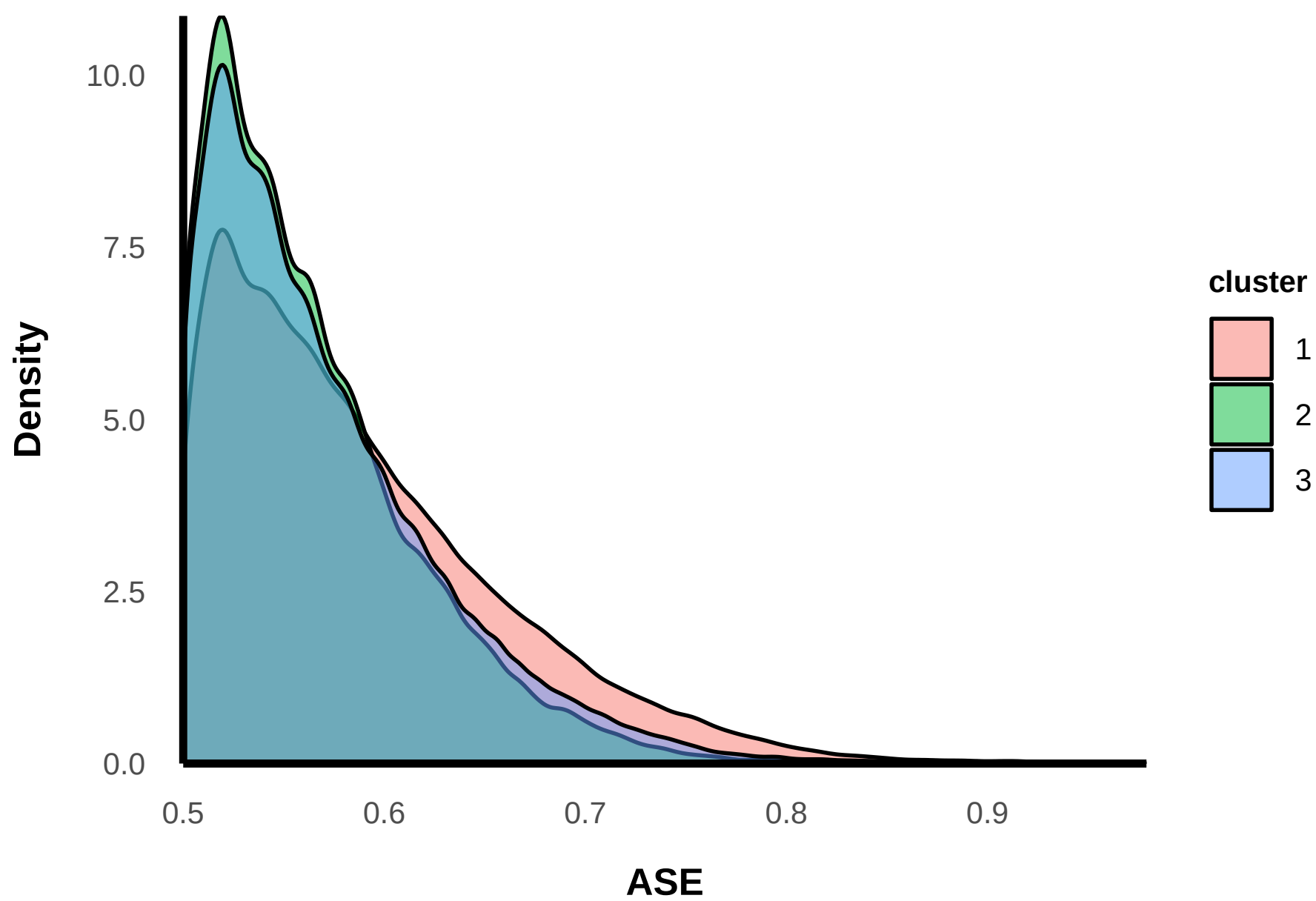

Supplementary Figure 4: Allele-specific expression scores distribution for cluster 1, cluster 2 and cluster 3.

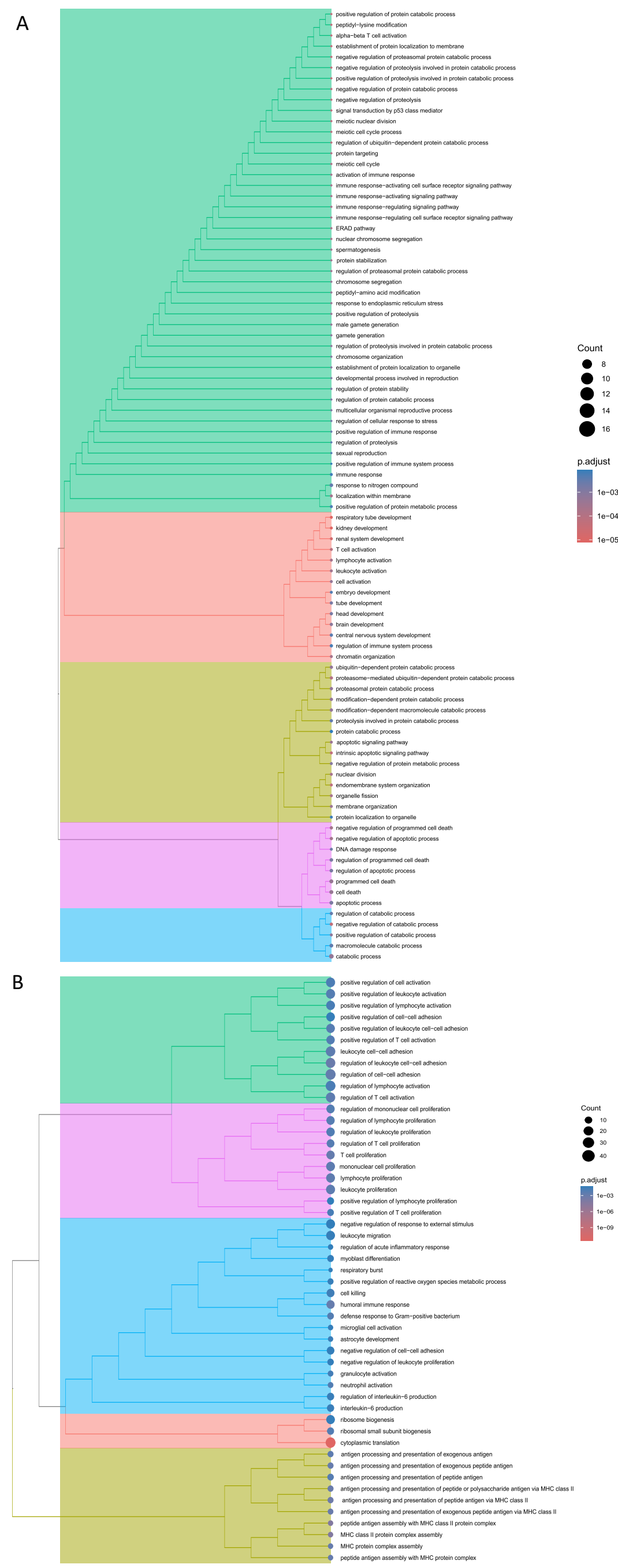

Supplementary Figure 5: Functional analysis of differential imbalanced and expressed genes between the across the three patient clusters. A) The treeplot shows the GO enrichment analysis result for the 36 differentially imbalanced genes. B) The treeplot shows the GO enrichment analysis result for the 723 differentially expressed genes across the three clusters (p-value < 0.05).

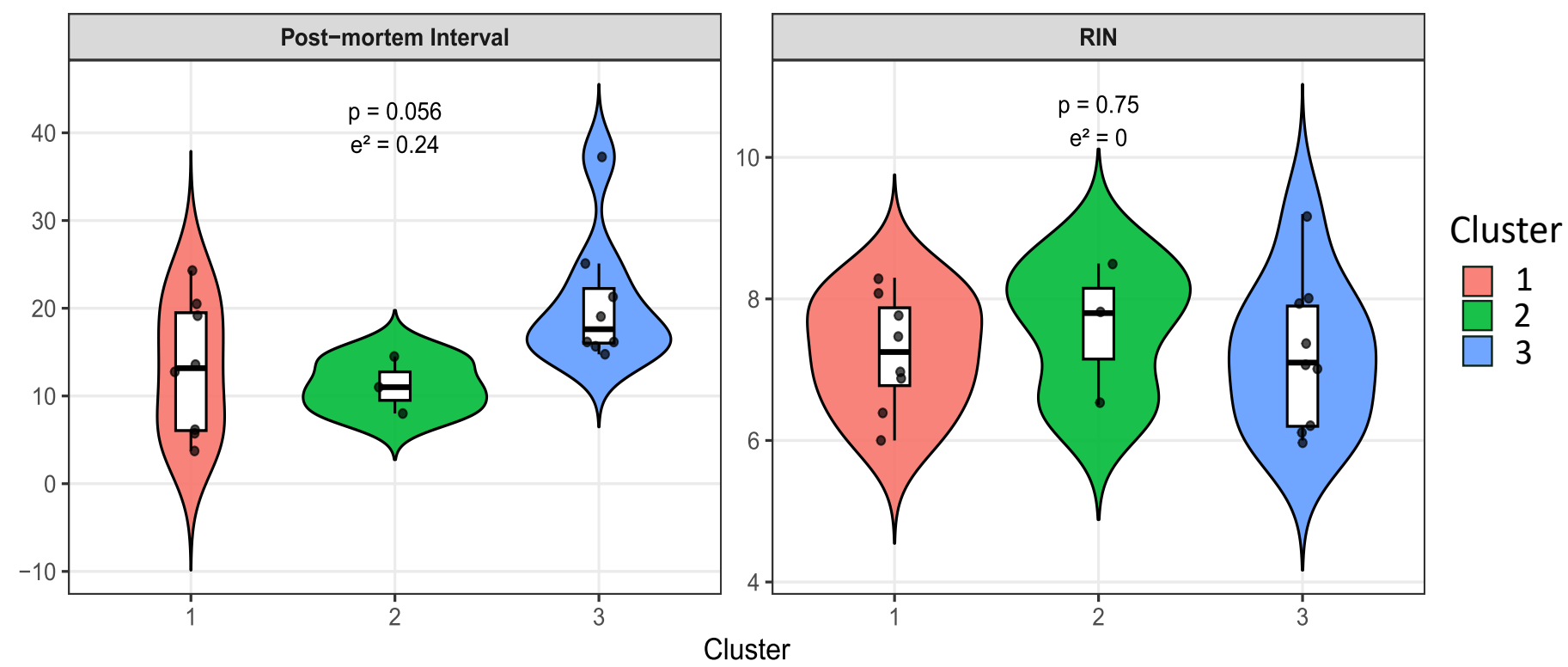

Supplementary Figure 6: Association of clinical variables with patient clusters. Violin plots showing the distribution of clinical parameters across the three patient clusters identified by network-based stratification. Variables shown include post-mortem internal and RNA Intergrity Number (RIN). Each violin plot shows the median and interquartile range. Nominal p-values and epsilon-squared (ϵ^2) effect sizes from the Kruskal-Wallis test are annotated for each variable. Clusters are color-coded: cluster 1 – 8 samples (red), cluster 2 – 3 samples (green), cluster 3 – 9 samples (blue).
